# Localization of Projectiles by Perspective Apparatus, Electrovibrators, Magnets, Etc.

**Published:** 1916-03

**Authors:** 


**Localization of Projectiles by Perspective Apparatus, Electrovibrators, Magnets, Etc.** The Archives of Medical Electrocity for several months, has devoted much space to various stereoscopic devices, finders of different types, including sextants, in accordance with general principles of perspective in connection with X-rays and to the other means mentioned. These journals may be consulted at the Grosvenor Library, Buffalo, our exchange file being sent there after review.

